# *GCH1* plays a role in the high-altitude adaptation of Tibetans

**DOI:** 10.24272/j.issn.2095-8137.2017.037

**Published:** 2017-05-18

**Authors:** Yong-Bo Guo, Yao-Xi He, Chao-Ying Cui, zhuluobu Ou, makangzhuo Bai, jizhuoma Duo, jiquzong De, ba Bian, Peng Yi, Cai-juan Bai, galanzi Gong, Yong-Yue Pan, la Qu, min Kang, yangji Ciren, mayangji Bai, Wei Guo, la Yang, Hui Zhang, Xiao-Ming Zhang, Wang-Shan Zheng, Shu-Hua Xu, Hua Chen, Sheng-Guo Zhao, Yuan Cai, Shi-Ming Liu, Wu Tian-Yi, Xue-Bin Qi, Bing Su

**Affiliations:** ^1^College of Animal Science and Technology, Gansu Agricultural University, Lanzhou Gansu 730070, China; ^2^State Key Laboratory of Genetic Resources and Evolution, Kunming Institute of Zoology, Chinese Academy of Sciences, Kunming Yunnan 650223, China; ^3^High Altitude Medical Research Center, School of Medicine, Tibetan University, Lhasa Tibet 850000, China; ^4^Kunming College of Life Science, University of Chinese Academy of Sciences, Kunming Yunnan 650204, China; ^5^Chinese Academy of Sciences Key Laboratory of Computational Biology, Max Planck Independent Research Group on Population Genomics, CAS-MPG Partner Institute for Computational Biology(PICB), Shanghai Institutes for Biological Sciences, Chinese Academy of Sciences, Shanghai 200031, China; ^6^Center for Computational Genomics, Beijing Institute of Genomics, Chinese Academy of Sciences, Beijing 100101, China; ^7^National Key Laboratory of High Altitude Medicine, High Altitude Medical Research Institute, Xining Qinghai 810012, China; ^8^School of Life Science and Technology, Shanghai Tech University, Shanghai 200031, China; ^9^Collaborative Innovation Center of Genetics and Development, Shanghai 200438, China

**Keywords:** *GCH1*, Positive selection, Tibetan, Hypoxia adaptation, Nitric oxide, Hemoglobin, Oxygen saturation

## Abstract

Tibetans are well adapted to high-altitude hypoxia. Previous genome-wide scans have reported many candidate genes for this adaptation, but only a few have been studied. Here we report on a hypoxia gene (*GCH1*, GTP-cyclohydrolase I), involved in maintaining nitric oxide synthetase (NOS) function and normal blood pressure, that harbors many potentially adaptive variants in Tibetans. We resequenced an 80.8 kb fragment covering the entire gene region of *GCH1* in 50 unrelated Tibetans. Combined with previously published data, we demonstrated many *GCH1* variants showing deep divergence between highlander Tibetans and lowlander Han Chinese. Neutrality tests confirmed a signal of positive Darwinian selection on *GCH1* in Tibetans. Moreover, association analysis indicated that the Tibetan version of *GCH1* was significantly associated with multiple physiological traits in Tibetans, including blood nitric oxide concentration, blood oxygen saturation, and hemoglobin concentration. Taken together, we propose that *GCH1* plays a role in the genetic adaptation of Tibetans to high altitude hypoxia.

## INTRODUCTION

Tibetans are a well-known example of successful adaptation to extreme environments at high-altitude. Compared to lowlanders moving to high altitude, Tibetans show greater lung capacity, function, diffusion, and ventilation, as well as lower hemoglobin (Hb) levels, better blood oxygen saturation, low hypoxic pulmonary vasoconstriction, high nitric oxide (NO) concentrations, and lower incidence of reduced birth weight (Beall, 2006; Beall et al., 1997; Erzurum et al., 2007; Wu & Kayser, 2006). These traits have been acquired during a long period of natural selection at high altitude after the ancestors of modern Tibetans permanently settled on the Qinghai-Tibetan Plateau during the early Upper Paleolithic period (Qi et al., 2013).

Various research groups have compared the genetic differences between Tibetan and Han Chinese using genome-wide scans, with two key genes identified (hypoxia-inducible factor 2α, *HIF2α*, also called *EPAS1*, and *EGLN1*) in the hypoxic pathway showing deep between-population divergence (Beall et al., 2010; Bigham et al., 2010; Peng et al., 2011; Simonson et al., 2010; Xu et al., 2011; Yi et al., 2010). For these two genes, Tibetan-specific haplotypes have been found, which are highly enriched in Tibetans (~80%), but rare or absent in other world populations (Lorenzo et al., 2014; Peng et al., 2011, 2017; Xiang et al., 2013). The function of selection on these two genes have been shown to cause blunted physiological responses under high altitude hypoxic conditions (Lorenzo et al., 2014; Peng et al., 2017; Xiang et al., 2013). For example, the Tibetan versions of *EPAS1* and *EGLN1* protect Tibetans from the over production of red blood cells (polycythemia), a common deleterious physiological response when lowlanders migrate to high altitude areas (Beall et al., 2010; Erzurum et al., 2007; Lorenzo et al., 2014; Peng et al., 2011, 2017; Petousi et al., 2014; Xiang et al., 2013; Xu et al., 2011; Zhang et al., 2010).

Many genes are likely involved in complex traits like high altitude adaptation. In addition to *EPAS1* and *EGLN1*, previous genome-wide studies have identified other candidate genes that might also contribute to genetic adaptation in Tibetans (Beall et al., 2010; Bigham et al., 2010; Peng et al., 2011; Simonson et al., 2010; Xu et al., 2011; Yi et al., 2010). The GTP-cyclohydrolase I (*GCH1*) gene is a reported candidate that shows relatively deep allelic divergence between Tibetans and Han Chinese, a sign of positive Darwinian selection (Peng et al., 2011). *GCH1* is located on human chromosome 14 (14q22.1-q22.2), spanning 60.8 kb with six exons. Furthermore, *GCH1* is a rate-limiting enzyme in the *de novo* synthesis of tetrahydrobiopterin (BH4). It has been reported that under hypoxia, *GCH1* can promote cancer growth, and its expression and that of endothelial nitric oxide synthetase (*eNOS*) is upregulated (Pickert et al., 2013). *GCH1* is considered as a major factor in maintaining nitric oxide synthetase (NOS) function and normal blood pressure, and its inhibition can increase blood pressure due to NOS uncoupling, which is found in many cardiovascular diseases such as hypertension and atherosclerosis (Antoniades et al., 2006). Hence, the known functional role of *GCH1* also makes it a candidate for high-altitude adaptation in Tibetans.

We resequenced an 80.8 kb fragment covering the entire gene region of *GCH1* in 50 unrelated Tibetans. Combined with published data, we found signals of positive selection on *GCH1* in Tibetans, with multiple sequence variants showing deep genetic divergence between highlander Tibetans and lowlander Han Chinese. Genetic association analysis detected significant correlation of the *GCH1* variants with multiple physiological traits of Tibetans, including blood nitric oxide (NO) concentration, blood oxygen saturation level, and hemoglobin concentration. Hence, *GCH1* might play an essential role in high-altitude adaptation in Tibetans.

## MATERIALS AND METHODS

### DNA sample collection and resequencing of GCH1 gene fragment

The 50 unrelated Tibetan samples were obtained from previous research (Peng et al., 2011). We resequenced an 80.8 kb fragment covering the gene region of *GCH1* and its flanking sequences (10 kb up-and down-stream of *GCH1*). For association analysis, we collected blood samples and extracted DNA from 226 unrelated adult Tibetans, whose physiological data were also collected with written informed consent. The protocol of this study was evaluated and approved by the Internal Review Board of Kunming Institute of China, Chinese Academy of Sciences.

### Detection of selection on GCH1 in Tibetans

The initially identified sequence variants were filtered by removing single nucleotide polymorphisms (SNPs) showing a significant deviation from the Hardy-Weinberg Equilibrium (HWE < 0.000 1), and those with an excessive missing genotype rate (MGR > 0.05). Four methods were used for selection testing, including two allele-frequency-based and two haplotype-based tests. Locus specific *F*_ST_ was calculated between 83 Tibetans and three reference populations (103 Han Chinese, CHB; 99 Europeans, CEU; and 108 Africans, YRI) following Weir & Cockerham (1984). The Tajima's *D*-test was also performed following the published procedure (Tajima, 1989).

The two haplotype-based tests included the iHS and XP-EHH tests (Sabeti et al., 2002). The iHS score was calculated for each site using selscan (Szpiech & Hernandez, 2014) based on the phased haplotypes, and only those allelic loci whose ancestral alleles were known with certainty were included in the analysis (Voight et al., 2006). The XP-EHH (Sabeti et al., 2007) analysis was used to detect the extended haplotypes due to positive selection. Han Chinese were used as the reference population in the XP-EHH test. We computed XP-EHH scores using selscan (Szpiech & Hernandez, 2014) based on phased haplotypes of Tibetans and Han Chinese. The XP-EHH score of each SNP was standardized by the mean XP-EHH and the standard deviation (*SD*) over the genome.

### Functional prediction of GCH1 candidate SNPs

Functional enrichment analyses of the candidate variants were performed using the Combined Annotation Dependent Depletion (CADD) database (<uritalic>http://krishna.gs.washington.edu/download/CADD/v1.3/1000G_phase3_inclAnno.tsv.gz</uritalic>), which incorporates data from ENCODE (<uritalic>http://genome.ucsc.edu/ENCODE/</uritalic>) and NIH Roadmap Epigenomics using ChromHMM (<uritalic>https://sites.google.com/site/anshulkundaje/projects/epigenomeroadmap#TOC-Core-Integrative-chromatin-state-maps-127-Epigenomes-</uritalic>) (<xref ref-type="bibr" rid="b7-ZoolRes-38-3-155">Ernst & Kellis, 2012</xref>).

Evolutionary constraint is an indication of functional importance. We used Genome Evolutionary Rate Profiling (GERP) to evaluate how conserved a test SNP was compared with SNP-containing sequences from different species (<uritalic>http://mendel.stanford.edu/SidowLab/downloads/gerp/</uritalic>). The GERP++ method was used to calculate site-specific RS scores (<xref ref-type="bibr" rid="b6-ZoolRes-38-3-155">Davydov et al., 2010</xref>). A positive GERP++ score indicates evolutionary constraint, and the greater the score, the greater the level of evolutionary constraint inferred to be acting on that site.

The H3K4Me1 value indicates the maximum ENCODE H3K4 methylation level (maximum value observed across 16 ENCODE cell lines at a given position), suggestive of an enhancer or other regulatory activities. The H3K4Me3 value indicates the maximum ENCODE H3K4 trimethylation level, and is an indication of a promoter. The DNase *P* value indicates evidence for open chromatin. The transcription factor binding site (TFBS) is indicated by the number of different overlapping ChIP transcription factor binding sites. In addition, the splice site results indicate if the tested variant is within an ACCEPTOR or a DONOR (Supplementary Table S1).

### Measurements of physiological traits

Physiological data and blood samples were collected from 226 unrelated Tibetans permanently residing in Bange county (*n*=135, 37.41±3.8 years old) at an elevation of 4 700 m and Lhasa city (*n*=91, 35.33±6.8 years old) at an elevation of 3 600 m. Written informed consent from all participants was obtained. For physiological parameters, we collected hemoglobin (Hb) concentration, arterial oxygen saturation (SaO_2_) level, and blood nitric oxide concentration, representing key adaptive physiological traits in Tibetans (Wu & Kayser, 2006).

The Hb level in blood was measured using a HemoCue Hb 201+ analyzer (Angelholm, Sweden). The SaO_2_ level was measured using fingertip blood with a hand-held pulse oximeter (Nellcor NPB-40, CA, USA). Blood NO was measured using a nitric oxide analyzer (Sievers Model-280, GE Analytical Instruments; Boulder, CO, USA).

### SNP genotyping and association analysis

We selected nine tag SNPs, covering the entire gene region of *GCH1* (Table 1). Genotyping was conducted by SNaPshot on an ABI 3130 sequencer (Applied Bio-Systems, Forster City, CA, USA). Genetic association analysis was executed using PLINK 1.07 (Purcell et al., 2007). We used an additive model in the association analysis as all candidate SNPs were non-coding and likely influenced the level of gene expression. Permutations (100 000 times for each test) were performed for statistical assessment and correction for multiple tests.

**1:** Association of nine *GCH1* variants with three physiological traits in Tibetans

Traits	SNP_ID	Male (*n*=91)		Female (*n*=135)		All (*n*=226)		
		Beta	EMP'	Beta	EMP'	Beta	EMP''	R^2^(%)
Hb	rs7148266	-4.33	0.48	-0.53	0.78	-1.51	0.78	0.05
	rs17128004	-3.02	0.65	-0.49	0.86	-1.10	0.86	0.01
	rs4411417	-4.33	0.48	-1.27	0.86	-1.85	0.69	0.23
	rs2183082	1.17	0.86	-2.10	0.57	-0.65	0.86	0.08
	rs10220344	1.17	0.86	-2.10	0.57	-0.65	0.86	0.08
	rs146540091	-62.95	0.05	-17.31	0.25	-35.15	0.02	1.65
	rs117863726	-62.95	0.05	-17.61	0.47	-36.27	0.02	2.39
	rs10136972	12.97	0.03	0.21	1.00	4.28	0.28	1.29
	rs112700866	22.04	0.05	-0.78	1.00	10.25	0.16	0.67
NO	rs7148266	-15.97	0.04	-10.82	0.04	-12.96	2.32E-03	3.05
	rs17128004	-17.23	0.03	-10.77	0.04	-13.42	2.32E-03	3.08
	rs4411417	-15.97	0.04	-11.67	0.04	-13.63	2.75E-03	3.76
	rs2183082	-6.35	0.54	-6.05	0.17	-6.32	0.08	1.28
	rs10220344	-6.35	0.54	-6.05	0.17	-6.32	0.08	1.28
	rs146540091	-162.70	3.53E-03	13.14	0.78	-41.65	0.07	3.36
	rs117863726	-162.70	3.53E-03	13.07	0.65	-41.05	0.07	1.40
	rs10136972	-5.71	0.65	-14.60	0.01	-11.35	0.02	1.88
	rs112700866	-10.98	0.35	4.97	0.73	-2.78	0.86	0.03
SaO_2_	rs7148266	-0.84	0.38	0.55	0.78	0.05	0.86	0.01
	rs17128004	-1.08	0.33	-0.05	1.00	-0.44	0.48	0.07
	rs4411417	-0.84	0.38	0.57	0.65	0.03	1.00	2.50E-03
	rs2183082	-0.50	0.57	-0.38	0.78	-0.44	0.52	0.03
	rs10220344	-0.50	0.57	-0.38	0.78	-0.44	0.52	0.03
	rs146540091	12.53	0.05	-1.61	0.78	3.07	0.54	3.22
	rs117863726	12.53	0.05	6.66	0.14	8.53	0.02	2.47
	rs10136972	-1.86	0.27	-1.84	0.11	-1.84	0.05	0.75
	rs112700866	-1.68	0.67	-3.46	0.17	-2.76	0.16	0.75
Hb, hemoglobin concentration; NO, blood nitric oxide concentration; SaO_2_, blood oxygen saturation level. EMP', *P* value after multiple test corrections; EMP'', *P* value after multiple test corrections with sex as the covariant.								

## RESULTS

### Resequencing of GCH1 in Tibetans and tests of selection

Previous DNA array-based genome-wide studies have only covered a limited number of *GCH1* sequence variants (Peng et al., 2011). To obtain complete sequence data of *GCH1*, we first resequenced an 80.8 kb fragment covering the entire gene region of *GCH1*(60.8 kb) as well as the flanking sequences (10 kb from upstream and 10 kb from downstream regions). In total, we sequenced 50 unrelated Tibetan individuals, as reported previously (Peng et al., 2011). In addition, we obtained the *GCH1* sequences of 33 Tibetans from recently published whole genome sequencing data (Lu et al., 2016), for a final sample size of 83.

We identified a total of 384 <italic>GCH1</italic> sequence variants (SNPs) in the 83 unrelated Tibetans, among which 245 were shared between Tibetans and the three lowland reference populations from the 1000 Genomes Project (<uritalic>http://www.1000genomes.org</uritalic>) (103 Han Chinese, CHB; 99 Europeans, CEU and 108 Africans, YRI). The remaining 139 SNPs were rare in Tibetans ( < 1.0%), and therefore not included in our analysis. The 245 SNPs were all located in the non-coding regions of <italic>GCH1</italic>.

**Figure 1:**
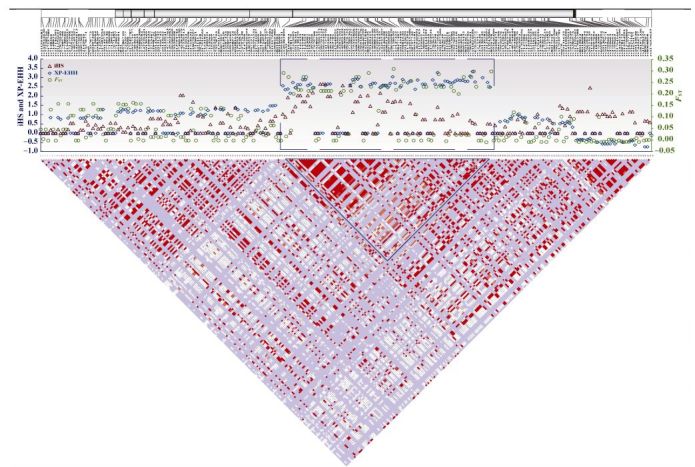
Genetic divergence of 245 *GCH1* variants between Tibetans and Han Chinese (*F*_ST_, iHS, and XP-EHH)

To detect whether *GCH1* was under selection in Tibetans, we conducted four different tests of selection, including two allele-frequency-based tests (*F*_ST_ and Tajima's *D*) and two haplotype-based tests (iHS and XP-EHH). We identified 49 *GCH1* SNPs with large between-population (Tibetan vs. Han) divergence (*F*_ST_ > 0.2), much larger than the genome average (*F*_ST_=0.03). These high-*F*_ST_ variants also showed high iHS and XP-EHH values (iHS > 0.2 and/or XP-EHH > 0.2) (Figure 1). They were aggregated in a relatively short region (7.5 kb) covering intron-1 and intron-2 of *GCH1*. These results suggest a clear signal of positive Darwinian selection on *GCH1* in Tibetans (Supplementary Table S1)

### Functional prediction and genetic association analysis

To determine the potential function of the SNPs with selection signals, we performed functional predictions using evolutionary constraint (GERP), transcription factor binding sites (TFBS), splicing motif, H3K4Me1/H3K4Me3 sites, and DNase-I hypersensitive sites. Results showed that there might be multiple functional sites, reflected by the consistent signals of different functional predictions (Supplementary Table S1).

**Figure 2:**
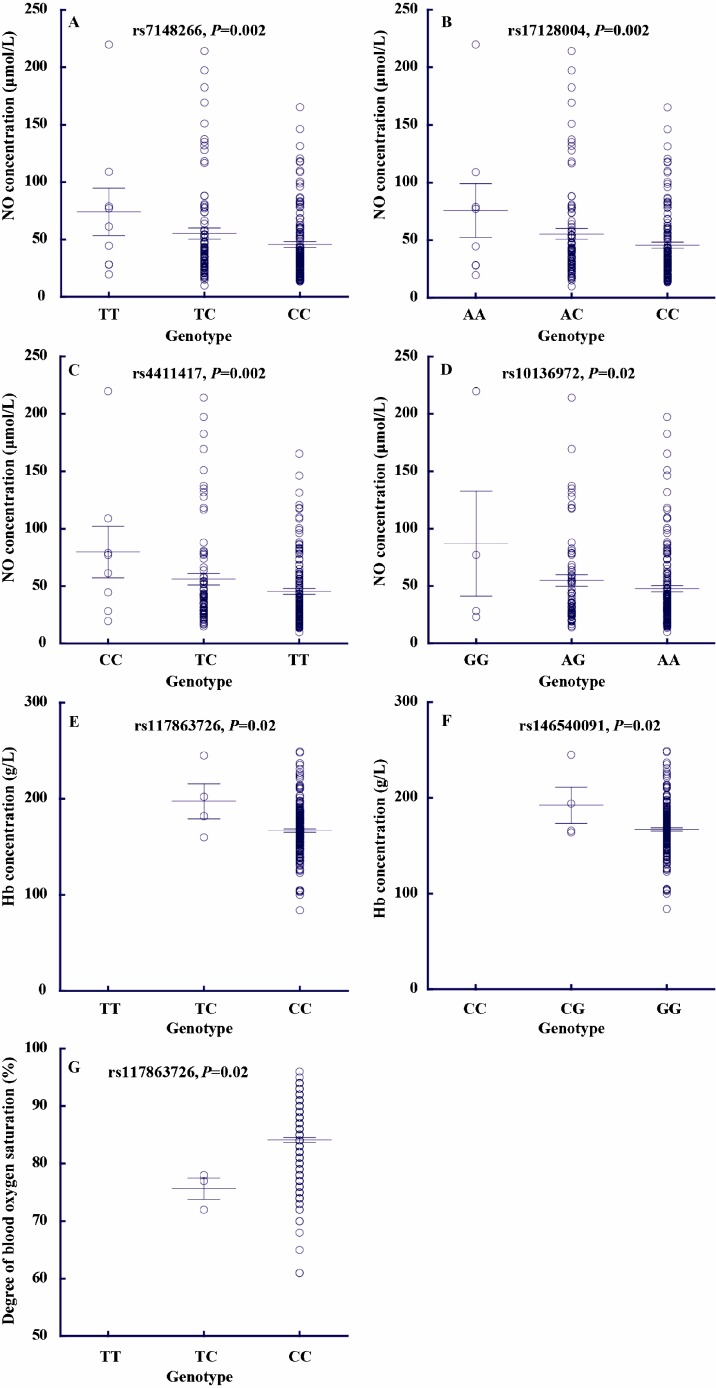
Genetic association of seven *GCH1* variants with three physiological traits (blood NO concentration, blood oxygen saturation level, and hemoglobin concentration)

To test whether these candidate *GCH1* variants contribute to the adaptive physiological traits of Tibetans, we measured blood NO concentrations, SaO_2_ levels, and Hb concentrations in 226 unrelated adult Tibetans (refer to methods for details). For genotyping, we selected nine tag SNPs to represent the entire gene region of *GCH1*. Notably, four SNPs (rs7148266, rs17128004, rs4411417, and rs10136972) showed significant association with blood NO levels, with the adaptive alleles exhibiting decreased NO. Each SNP accounted for 1.88%-3.76% of NO variance (Figure 2). Similar results were observed when males and females were analyzed separately (Table 1). Additionally, two SNPs (rs146540091 and rs137863726) showed association with hemoglobin concentration, with the adaptive alleles having lower Hb levels, consistent with previous results for *EPAS1* and *EGLN1* (Peng et al., 2011, 2017; Xiang et al., 2013). Another SNP (rs137863726) was associated with blood oxygen saturation, with the adaptive allele exhibiting higher SaO_2_ levels.

For the four SNPs (rs7148266, rs17128004, rs4411417, and rs10136972) showing significant association with blood NO levels, functional prediction analysis indicated that they were located in the *GCH1* intron regions with peak signals for H3K4Me1, H3K4Me3, DNase-I, and TFBS. For example, the H3K4Me1 peak values for rs10136972 and rs4411417 were 8.2 and 5.9, respectively, indicating their potential role in gene expression regulation of *GCH1*.

## DISCUSSION

Hypoxia serves as a key stress in high altitude environments. For lowlanders, prolonged exposure to high altitude hypoxia can cause chronic mountain sickness, reflected by the over production of red blood cells (polycythemia) as well as other deleterious physiological changes (Hackett & Roach, 2001; Macinnis et al., 2010). Tibetans are genetically adapted to high altitude hypoxia, and exhibit blunted physiological responses, e.g., relatively low hemoglobin levels (Lorenzo et al., 2014; Peng et al., 2011, 2017; Xiang et al., 2013). Key hypoxic pathway genes *EPAS1* and *EGLN1* were reported to be responsible for these blunted physiological responses (Lorenzo et al., 2014; Peng et al., 2017; Xiang et al., 2013). However, while the Tibetan version of these two genes provide protection against polycythemia, they do not explain all physiological changes in Tibetans, suggesting there might be other genes involved given the complexity of high altitude adaptation.

Based on resequencing and population analysis, we confirmed a signal of selection on *GCH1* in Tibetans. We identified more than 40 variants showing deep allelic divergence between highlander Tibetans and lowlander Han Chinese, with some having potential functional effects based on prediction using ENCODE. *GCH1* is a rate-limiting enzyme, acting as a crucial factor for maintaining normal NO synthetase function and blood pressure. Inhibition of *GCH1* activity is related to several cardiovascular diseases, with *GCH1* found to prevent hypoxia-induced pulmonary hypertension (Khoo et al., 2005). Hence, the function of selection on *GCH1*in Tibetans is expected to help maintain proper cardiovascular function at high altitude.

Hypoxic pulmonary vasoconstriction and pulmonary vascular structural remodeling are dominant pathophysiological characteristics of hypoxic pulmonary hypertension (Galiè et al., 2016; McLaughlin & McGoon, 2006; Schermuly et al., 2011). When lowlanders move to high altitudes, pulmonary hypertension usually occurs within a few weeks (Wilkins et al., 2015; Wu & Kayser, 2006); however, Tibetans rarely develop this condition. We showed that *GCH1* SNPs were associated with NO levels in the blood. *GCH1* is involved in the synthesis of tetrahydrobiopterin (BH4), a vital regulator of eNOS, the endothelial-form enzyme producing NO, an important molecule for vasodilation, which is considered the main reason for the superior blood flow and pulmonary pressure in Tibetans (Pickert et al., 2013). In hph-1 mice, deficiency of BH4 causes hypoxia-induced pulmonary hypertension even under normoxic conditions (Khoo et al., 2005). The overexpression of *GCH1* in mice could prevent hypoxia-induced pulmonary hypertension due to the augmentation of BH4 (Khoo et al., 2005). Hence, it is possible that *GCH1* regulates pulmonary vasoconstriction responses in Tibetans by influencing NO production in the blood. We observed four *GCH1* variants showing significant association with blood NO levels. As these variants are located in the *GCH1* intron regions with peak enhancer and/or promoter activity signals, they are likely involved in the regulation of *GCH1* expression and eventually affect blood NO production, which needs further investigation. We also observed associations of *GCH1* SNPs with oxygen saturation and hemoglobin; however, the underlying molecular mechanisms are yet to be studied.

In summary, we demonstrated that *GCH1* has been under positive selection in Tibetans. We identified many variants with deep allelic divergence between Tibetans and lowlanders. The association and known function results suggest the potential involvement of *GCH1* in the regulation of multiple physiological traits in Tibetans.

## ACKNOWLEDGEMENTS

We are grateful to all the volunteers participated in this study.
